# Prevalence and determinants of poor outcome in patients with chronic kidney disease in low-income countries in sub-Saharan Africa: a systematic review

**DOI:** 10.11604/pamj.2026.53.168.44897

**Published:** 2026-04-20

**Authors:** Chitalu Chanda, Nomanthombi Johnson, Natasha Ngwenya, Christabel Silavwe, Mukuka Chibamba, Eustarckio Kazonga

**Affiliations:** 1 Department of Public Health, School of Medicine and Health Sciences, University of Lusaka, Lusaka, Zambia,; 2 Department of Public Health, Mulungushi University, Livingstone, Zambia

**Keywords:** Chronic kidney insufficiency, prevalence, mortality, sub-Saharan

## Abstract

Chronic kidney disease (CKD) is a significant health burden globally, associated with high healthcare costs and mortality rates. In Africa, the prevalence of CKD is estimated at around 10-15% in adults, with factors such as HIV, diabetes mellitus, hypertension, and cardiovascular disease contributing to its prevalence. We performed a systematic review to evaluate the prevalence of chronic kidney disease in sub-Saharan Africa between 2018 and 2023. We searched Google Scholar, PubMed, and Cochrane Review from 2018 until 2023, and we included studies that looked at the prevalence of chronic kidney disease. The secondary outcome of interest was patient as defined by any cause of mortality. A comprehensive search yielded 10 studies for analysis, reporting a prevalence rate of CKD ranging from 9.8% to 29.9%. Factors such as age, hypertension, and diabetes mellitus were found to be associated with CKD prevalence. A total of 6,963 patients were involved in the studies. However, none of the included studies explored the outcome of mortality among CKD patients. The study was found to have a good assessment using the Ottawa-Newcastle scoring for cross-sectional studies. The prevalence of chronic kidney disease is high. The outcomes of chronic kidney disease are poorly explored in sub-Saharan Africa.

## Introduction

Chronic Kidney Disease (CKD) is associated with significant healthcare costs, morbidity, and mortality [1,2]. Globally, the prevalence of chronic kidney disease is estimated at around 10% [1,3]. On the African continent, the prevalence of chronic kidney disease was approximately between 10 and 15% among adults [4,5]. Chronic kidney disease is often associated with other conditions such as Human Immunodeficiency Virus (HIV), use of non-steroidal anti-inflammatory drugs, diabetes mellitus, hypertension, viral hepatitis, and cardiovascular disease [1,6,7]. There has been an increase in the prevalence of non-communicable conditions such as hypertension, diabetes mellitus and obesity in Africa [8]. Even among HIV-positive patients, whose prevalence is highest in Africa, the incidence of non-communicable diseases has greatly increased [9,10].

The epidemiological transition of CKD in Africa has been documented in remote and rural areas [11]. This could be influenced by the ability to diagnose the condition through increased testing of markers of chronic kidney disease in Africa. Furthermore, the prevalence could be influenced by increased risk factors such as obesity, hypertension, and diabetes mellitus in Africa [12]. The morbidity usually associated with chronic kidney disease is poor quality of life and increased health care costs. The outcome of mortality among chronic kidney disease patients is influenced by age, stage of kidney disease, anaemia, hemodialysis, comorbidity conditions, and renal replacement therapy [1,2,13,14]. Therefore, the study sought to explore the prevalence of chronic kidney disease as well as factors associated with outcomes in sub-Saharan Africa.

The research question was: what is the prevalence and factors associated with chronic kidney disease in sub-Saharan Africa? The objectives of this study were to investigate the prevalence of chronic kidney disease among adults in sub-Saharan Africa, to examine the factors associated with the prevalence of chronic kidney disease in sub-Saharan Africa and to explore the outcomes associated with mortality among patients with chronic kidney disease in sub-Saharan Africa

## Methods

The Preferred Reporting Items for Systematic Reviews and Meta-Analysis (PRISMA) guidelines were used to guide the review of articles, and the quality of the studies was measured and assessed using the Ottawa-Newcastle scoring tool adapted for cross-sectional studies.

**Data sources and search strategy:** we conducted a comprehensive search across multiple databases, including Google Scholar and PubMed, using key search words such as “Mortality” OR “Rate” AND “Outcome” AND “Chronic Kidney Diseases” AND “Factors Associated” AND “Low-Income Countries (LMIC)” AND “sub-Saharan Africa” as shown in Table 1. The search encompassed studies conducted from 2018 to 2023. The search was conducted and verified before outcomes were input into Rayyan, an online platform for data screening and eligibility analysis.

**Table 1:** search characteristics

Chitalu Chanda (CC)	Natasha Ngwenya (NN)	Nomanthombi Johnson (NJ)	Christabel Silavwe (CS)	Mukuka Chibamba (MC)	Eustarckio Kazonga (EK)
("Chronic kidney insufficiency") AND prevalence AND outcomes OR Dialysis AND Africa	Prevalence of Chronic Kidney Disease CKD AND Outcomes OR risk Factors in sub-Saharan Africa	(Prevalence) AND “Mortality” OR Rate AND Outcome AND Chronic Kidney diseases OR Chronic Kidney Insufficiency AND “factors associated” AND Low-Income countries OR LMIC AND sub-Saharan Africa	Chronic kidney disease; sub-Saharan Africa; low-income countries; mortality; prevalence	Chronic kidney disease; sub-Saharan Africa; low-income countries; mortality; epidemiology	Verified all searches

**Search words:** (Prevalence) AND “Mortality” OR Rate AND Outcome AND Chronic Kidney Diseases AND “Factors Associated” AND Low-Income Countries OR LMIC AND sub-Saharan Africa. (Chronic Kidney Insufficiency) AND Prevalence AND Outcomes OR Dialysis AND Africa. (Prevalence) AND “Mortality” OR Rate AND Outcome AND Chronic Kidney Diseases OR Chronic Kidney Insufficiency AND “Factors Associated” AND Low-Income Countries OR LMIC AND sub-Saharan Africa. (Prevalence) AND “Mortality” AND Outcome AND (Dialysis*) AND Chronic Kidney Diseases OR Chronic Kidney Insufficiency AND “Factors Associated” AND Low-Income Countries OR LMIC AND sub-Saharan Africa. (“Chronic Kidney Insufficiency”) AND Prevalence AND Outcomes AND Dialysis AND Africa. Prevalence of Chronic Kidney Disease CKD AND Outcomes OR Risk Factors in sub-Saharan Africa

**Selection criteria:** the selection criteria included all studies that all published articles had reported; i) participants who were 15 years and above; ii) diagnosis of chronic kidney disease was established as defined by creatinine clearance of less than 65 mmol/hr; iii) all observations, cross-sectional, randomized control studies and case-controls were included in the analysis that reported prevalence and/or associated factors for the chronic kidney disease (iv) Studies done in sub-Sahara Africa (iv) within 5 years of publication. The following exclusion criteria were used during the screening and review of the search: (i) all studies in languages that were English; ii) abstracts and conference posters; iii) publications that were beyond 10 years; iii) studies that were not done in sub-Saharan Africa; iv) review articles and case series will be excluded. All titles and/or abstracts were examined by at least two reviewers, with a minimum of two reviewers agreeing to include the article. EK verified the decisions. Those not meeting the criteria after reviewing the abstract and title were excluded. If there was a conflict of decision regarding the articles, a third reviewer was asked to review and decide. After the first evaluation and screening, all the remaining articles were fully read, and the studies meeting the inclusion criterion were included in the systematic review. Disparities were resolved through discussion.

**Data extraction:** the reviewers participated in the data extraction from the included articles. The following information was extracted: Journal title/name, name of author, year of publication, study design, number of participants, the prevalence of chronic kidney disease, stage of kidney disease, Odds Ratio or Relative Risk or Hazard Ratio for risk factors and mortality (outcome). The data were extracted using Rayyan and Excel.

**Quality assessment:** the quality of the studies was assessed using the Newcastle-Ottawa scoring tool, which is attached in the Supplementary appendix (Table 2) [15]. The grading system followed for the Newcastle-Ottawa score was: (0-3) poor quality, (3-5) Fair quality and (6-9) good quality. All articles scored between 6 and 9 and were classified and rated as good quality.

**Table 2:** assessment of the quality of the study using Newcastle-Ottawa adapted for a cross-sectional study

Author Surname Name	Year of Publication	Selection				Comparability		Outcome	Score
		Representativeness of the cases	Sample size	Non-Response rate	Ascertainment of the screening/surveillance tool	The potential confounders were investigated by subgroup analysis or multivariable analysis.	Assessment of the outcome	Statistical test	
Kebede *et al*.	2022	*	*	*	**	*	**	*	9
Ploth *et al*.	2019	*			**		**	*	6
Fiseha *et al*.	2019	*	*	*	**	*	**	*	9
Ali *et al*.	2023	*	*	*	**	*	**	*	9
Sertsu *et al*.	2022	*	*	*	**	*	**	*	9
Kachimanga *et al*.	2021	*		*	**	*	**	*	8
Bahrey *et al*.	2020	*			**	*	**	*	7
Kaze *et al*.	2021	*	*		**	*	**	*	8
Tannor *et al*.	2019	*	*	*	**	*	**	*	9
Alemu *et al*.	2020		*	*	**	*	*	*	7

The (*) represents a single score for each domain assessed in the studies reviewed. These are then summed up to give the row total for the overall quality of the studies. (*) is equivalent to a score of 1, and (**) is equivalent to a score of 2.

## Results

**Figure 1:**
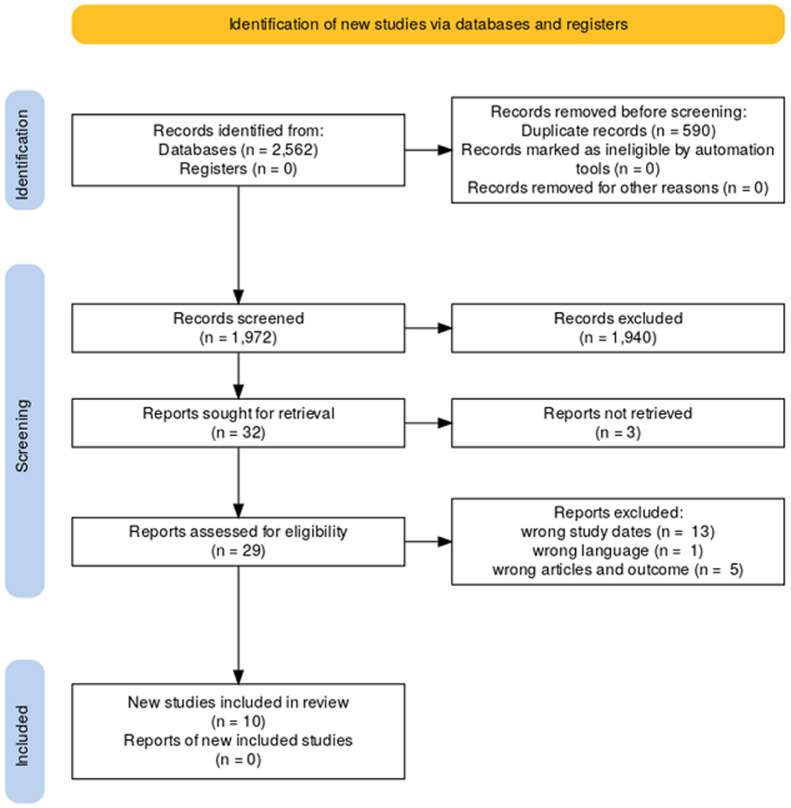
PRISMA flow diagram

**Study selection:** the search retrieved 2,562 articles, of which 32 were selected for full-text review. Of these eligible studies, 10 were included for data abstraction, as shown in the PRISMA diagram in Figure 1.

**Characteristics of studies included:** ten studies were included in the analysis. Table 3 highlights the features of the studies included. The total number of participants was 6,963. All 10 studies were cross-sectional. The majority of studies were conducted in one country (six), while others were distributed across the other regions in sub-Saharan Africa. All the articles included had a good assessment with the Newcastle-Ottawa assessment tool.

**Table 3:** study characteristics

Author's last name	Study type	Publication year	Study Country	Number of participants
Bahrey *et al*.	cross-sectional	2019	Ethiopia	578
Ploth *et al*.	cross-sectional	2018	Tanzania	712
Kebede *et al*.	cross-sectional	2022	Ethiopia	326
Kaze *et al*.	cross-sectional	2021	Cameroon	433
Tannor *et al*.	cross-sectional	2019	Ghana	2781
Fiseha *et al*.	cross-sectional	2019	Ethiopia	414
Kachimanga *et al*.	cross-sectional	2021	Sierra Leone	318
Ali *et al*.	cross-sectional	2023	Ethiopia	509
Sertsu *et al*.	cross-sectional	2022	Ethiopia	620
Alemu *et al*.	cross-sectional	2020	Ethiopia	272

**Study characteristics:** the 10 studies were carried out in a variety of geographic locations: Ethiopia (n=6), Tanzania, Sierra Leone, Cameroon, and Ghana (n=1 in each country). All 10 included articles represented cross-sectional studies.

**Study setting:** a total of 6,963 participants were studied in the systematic review across 10 articles. Most of these participants were recruited in a health facility (clinic or hospital), as depicted in 7 out of 10 articles (70%) and the rest were recruited in a community-based setting, as depicted in 3 out of the 10 articles (30%).

**Prevalence:** the primary outcome of interest was the prevalence of chronic kidney disease. The study definition of chronic kidney disease was renal creatinine clearance of less than 65 mmol/hr. The prevalence rate ranged from 9.8 to 29.9% as shown in Table 4 [1,4,16-22]. None of the 10 studies that were included explored the outcome of mortality among the patients. There was no report of associated factors to the poor outcome.

**Table 4:** prevalence and associated factors of chronic kidney disease in sub-Sahara Africa

Author	Study type	Publication year	Study Country	number of participants	Prevalence of CKD	odds of CKD prevalence by age	The odds ratio for Diabetes mellitus about the prevalence of CKD	The odds ratio for hypertension	Odds) Association of CKD prevalence and cardiac disease	level of the scoring of the study
Bahrey *et al*.	Cross-sectional	2019	Ethiopia	578	22.10%	1.43CI 1.07,1.81)	2.13 CI 1.07,4.26)	9.45CI 1.34,14.73)	not reported	`7/9
Ploth *et al*.	Cross-sectional	2018	Tanzania	712	12.4% CI 10.2, 14.8)	Not reported	Not reported	Not reported	not reported	`9/9
Kebede *et al*.	Cross-sectional	2022	Ethiopia	326	7.40%	Not reported	Not reported	1.5	not reported	9/9
Kaze *et al*.	Cross-sectional	2021	Cameroon	433	11.70%	not reported	Not reported	Not reported	Not reported	8/9
Tannor *et al*.	Cross-sectional	2019	Ghana	2781	28.50%	Not reported	not reported	1.02	not reported	9/9
Fiseha *et al*.	Cross-sectional	2019	Ethiopia	414	7.7% - 16.9%	AOR 10.8	0.0963	0.1188	not reported	`9/9
Kachimanga *et al*.	Cross-sectional	2021	Sierra Leone	318	29.90%	40-60 years = OR 0.7333; 60 and above = OR 1.5667	0.1556	2.4667	Not reported	8/9
Ali *et al*.	Cross-sectional	2023	Ethiopia	509	21.30%	40-60 vs. 15-39 OR = 0.7333; 60years and above vs. 15-39 years OR = 1.5667	2.1176	1.3088	Not reported	`7/9
Sertsu *et al*.	Cross-sectional	2022	Ethiopia	620	9.8-22-4%	Above 60 years OR = 1.83 as compared to those Below 60 years	2.43	4.61	2.55	`9/9
Alemu *et al*.	Cross-sectional	2020	Ethiopia	272	17.30%	1.3CI 0.5,2.9)	0.8 CI 0.2,1.2)	6CI 4,22)	2 CI 0.9,5)	7/9

**Effect of age:** the ages of the participants were 18 years and above. Prevalence of CKD increased with age. Two (2) studies out of 10 (20%) (Kachimanga *et al*.; Ali *et al*.) compared the odds of CKD prevalence by age, comparing those 40-60 years to those above 60 years. The mean odds ratio for those 40-60 years was 0.7333 compared to the odds ratio of those above 60 years, which was 1.566. This indicated that those above 60 years had a higher likelihood of developing CKD compared to those below 60 years. Another study with 620 participants (Sertsu *et al*.) [20-22] compared the odds of CKD prevalence between those below 60 years and those above 60 years. The odds of CKD prevalence in those above 60 years were 1.83 compared to those below 60. This study also indicated that the prevalence of CKD increases with age.

**Effect of hypertension and diabetes on CKD:** in this systematic review, the major comorbidities were DM and HTN, which were correlated with CKD with varying odds ratios. Six (6) out of 10 articles (60%) reported odds ratios for diabetes mellitus about CKD ranging from 0.0963 to 2.13. Three (3) out of the 6 articles (50%) reported a reduced odds ratio of diabetes about CKD, while the other 50% of the articles reported twice as likely for diabetics to develop CKD [1,19-23]. Poorly controlled diabetes increases the chances of developing CKD. Eight out of 10 articles (80%) reported odds ratios of hypertension about CKD ranging from 0.001188 to 9.45. Only 1 article of the 8 (12.5%) had a reduced odds of hypertension associated with CKD, 7 out of 8 (87.5%) showed an increased likelihood of CKD in the presence of hypertension. Hypertension, therefore, increases the chances of developing CKD. Only 1 article reported COVID-19 in CKD, and there was no report on any association with CKD. There was only one article that reported the effect of HIV on CKD, with the association not being statistically significant.

## Discussion

This systematic review included 10 studies in which all the studies reported the prevalence of chronic kidney disease. The reported range of prevalence was higher than previously shown in some studies [6,12,19,24]. The included studies showed a reduced risk of bias as demonstrated by the Newcastle-Ottawa assessment score.

The studies included showed a heterogeneous distribution of the comorbid conditions related to chronic kidney disease. Most of the articles included did not include COVID-19 among the variables of interest and impact. Only one paper refers to COVID-19. As seen from previous studies, the risk of chronic kidney disease was associated with other non-communicable diseases such as diabetes mellitus and hypertension [12,20,25].

Only one study explored the association between chronic kidney disease and HIV despite the prevalence of HIV being higher in sub-Saharan Africa [20]. Among the associated risk factors, at least 6 studies showed that hypertension was associated with chronic kidney disease [18,20-23,26]. However, this association does not highlight causality between hypertension and chronic kidney disease. The increased coexistence of the comorbid conditions might have a significant impact on polypharmacy, health costs, and quality of life [2]. This can be seen as some studies reported increased use of non-steroidal anti-inflammatory drugs, which might worsen kidney function. Age and increased body mass index were reported in some studies as being associated with CKD [18]. These could indicate that routine screening can be recommended in those of increased age for non-communicable diseases.

Three studies reported cardiovascular disease, with a statistically significant association being demonstrated [18,20,22]. The mortality related to CKD and cardiovascular disease has been previously shown [24]. Only one article reported COVID-19 in CKD, and there was no report on any association with CKD. No article reported the effect of HIV on CKD. However, there is an association with severe forms of COVID-19 in those with CKD [27,28]. The COVID-19 pandemic has recently affected many regions of Africa. One of the observations was reduced health facility attendance and reduced service provision during the pandemic [29,30]. However, COVID-19 was associated with increased unfavourable outcomes in those with comorbid conditions such as diabetes mellitus and hypertension, which are increased among patients with chronic kidney disease [27].

**Outcome of CKD:** none of the studies looked at the secondary outcome of mortality or its associated factors. No article reported outcomes relating to anaemia, infections, heart failure, or mortality. A previous systematic review, including data from 1990 to 2017, showed that all-cause mortality was 4.6% [24].

**Limitations:** conducting studies on chronic kidney disease (CKD) during the COVID-19 pandemic presented several challenges. In several situations, access to SARS-CoV-2 testing differed dramatically between patient groups. Furthermore, baseline renal function data were frequently missing, making it difficult to appropriately diagnose CKD and acute kidney damage in several trials. Researchers encountered problems due to variations in virus prevalence, targeted public health programmes, and vaccination rates. These differences influenced the incidence of SARS-CoV-2 infection throughout time and place, thus affecting the generalizability of findings of its impact on patients with chronic kidney disease. The excluded trial due to language could have had some significant findings which could not be interpreted.

## Conclusion

This review has shown and supported that the prevalence of chronic kidney disease is high in sub-Saharan Africa. Other non-communicable diseases, such as hypertension, cardiovascular disease, and diabetes, are significantly associated with chronic kidney disease. The outcomes of chronic disease remain poorly described in sub-Saharan Africa.

### What is known about this topic

- The prevalence of CKD was estimated to be 10 to 15;
- Mortality related to CKD was relatively high;
- The association of CKD with NCD.

### What this study adds

- The review highlights the populations and distribution of CKD in sub-Saharan Africa with increasing prevalence compared to the previous report;
- It shows increasing mortality reported;
- Furthermore, it gives insights into what the impact of COVID-19 might be.

## Annex

**Annex 1**: supplementary material (PDF 257 KB)
